# P015 Long-standing isolated alkaline phosphatase elevation attributed to vitamin D deficiency revealing extensive polyostotic Paget’s disease of bone: a diagnostic challenge and therapeutic dilemma

**DOI:** 10.1093/rap/rkag113.045

**Published:** 2026-11-03

**Authors:** Tony Yap, Erica Gurung, Mohammed Chaudhry, Nadia Ahmad

**Affiliations:** Basingstoke and North Hampshire Hospital, Basingstoke, United Kingdom; Basingstoke and North Hampshire Hospital, Basingstoke, United Kingdom; Basingstoke and North Hampshire Hospital, Basingstoke, United Kingdom; Basingstoke and North Hampshire Hospital, Basingstoke, United Kingdom

## Abstract

**Introduction:**

Paget’s disease of bone (PDB) is a chronic metabolic bone disorder characterised by accelerated and disorganised bone remodelling. It predominantly affects adults over 55 years of age and ranges from asymptomatic monostotic disease to extensive polyostotic involvement with significant skeletal complications. Diagnosis relies on characteristic biochemical and imaging findings, with persistent elevation of serum alkaline phosphatase (ALP) often providing the first clue. Intravenous bisphosphonates, particularly zoledronic acid, remain the treatment of choice when indicated.

This case highlights delayed diagnosis following prolonged attribution of raised ALP to vitamin D deficiency and the ongoing controversy surrounding treatment of asymptomatic extensive PDB.

**Case description:**

We present a healthy and active Caucasian gentleman in his mid-seventies with a background of hypertension, pre-diabetes and right thumb osteoarthritis. He is an ex-smoker and drinks moderate amounts of alcohol.

He was initially referred to rheumatology for persistently isolated raised ALP of 400U/L (40-129U/L), along with raised ALP bone isoenzyme. This has been the case for the past 13 years and was treated as Vitamin D deficiency. Recent family history interviewing transpired that he has a positive family history of Paget’s disease in his maternal side of the family in every generation.

He denied any musculoskeletal symptoms, bony pain, hearing loss, bony deformities, previous fractures nor constitutional symptoms. There had been no significant changes to his medications. Examination of his joints, spine and cardiorespiratory system were normal.

Biochemically, he had normal liver function including GGT and renal function. His parathyroid hormone, thyroid hormone, immunoglobulins, calcium, phosphate and inflammatory markers were normal. Liver autoimmune screen and coeliac screen were negative. His Vitamin D levels were slightly low at 41 nmol/L (>50 nmol/L). Further ALP isoenzyme testing showed predominance of bone ALP.

He had a mildly raised PSA. A digital rectal exam revealed smooth enlarged prostate. He denies any urinary symptoms and urinalysis was normal. He was referred to urology who suggested trial of tamsulosin and finasteride.

A whole-body isotope bone scan was performed showing increased uptake in the left femur, left hemipelvis, right ischium along with the thoracic vertebra and right calcaneus (Image 1). This was indicative of polyostotic Paget’s disease.

He was given a single dose of 5mg IV zoledronic acid infusion with a plan to monitor his ALP levels.

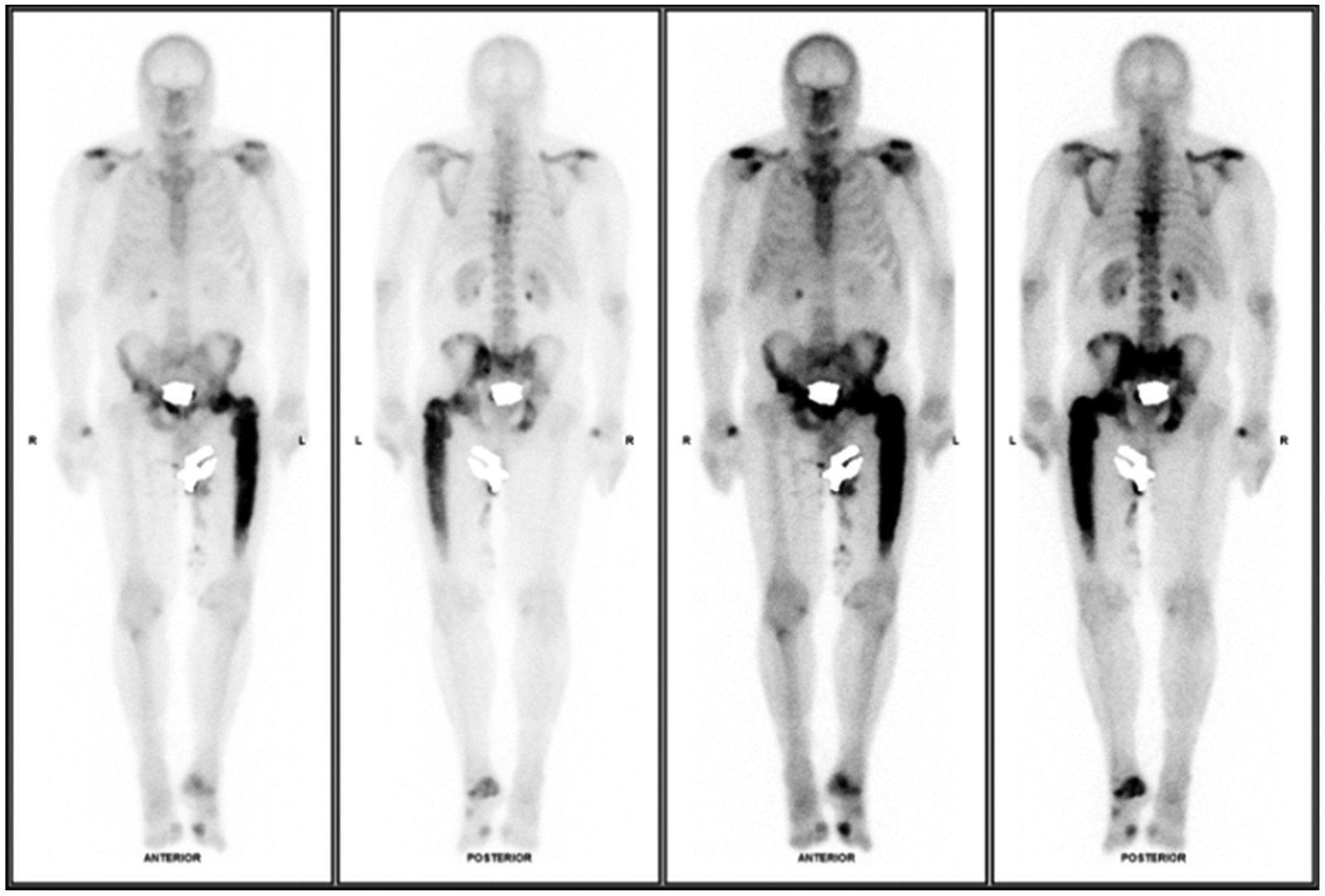

P015 Image 1: Bone isotope scan showing increased tracer uptake in left femur, left hemipelvis and right ischium appearing expanded. Further uptake in thoracic vertebrae and left calcaneus.

**Discussion:**

This case highlights two important clinical challenges: delayed recognition of Paget’s disease of bone (PDB) and uncertainty regarding treatment of extensive asymptomatic disease.

The patient had persistently elevated bone-specific alkaline phosphatase for over a decade, yet this was repeatedly attributed to mild vitamin D deficiency. Although vitamin D deficiency may contribute to elevated ALP, persistently isolated elevation despite replacement should prompt investigation for alternative causes of increased bone turnover, including PDB, particularly when liver biochemistry is normal. Earlier recognition of the strong multigenerational family history may also have increased diagnostic suspicion.

All major guidelines recommend serum alkaline phosphatase as the initial biochemical investigation for suspected PDB, with plain radiographs to confirm characteristic changes and radionuclide bone scintigraphy to determine disease extent. In this case, bone scintigraphy demonstrated extensive polyostotic involvement of the pelvis, femur, thoracic spine and calcaneus despite the absence of symptoms.

The management of asymptomatic PDB remains controversial. The 2019 UK Clinical Guideline, informed by the PRISM trial, recommends a symptom-directed approach, advising bisphosphonates primarily for Paget-related bone pain because intensive biochemical normalisation did not reduce fractures, orthopaedic surgery or improve quality of life. In contrast, the 2014 Endocrine Society guideline recommends treating metabolically active disease in patients at risk of future complications, particularly when high-risk sites such as the spine, weight-bearing bones or bones adjacent to major joints are involved. Similarly, the 2024 Italian SIOMMMS position paper supports treatment of symptomatic patients and favours treatment of most newly diagnosed asymptomatic patients with active disease, especially with polyostotic or high-risk skeletal involvement. Given this patient’s extensive disease affecting the femur, pelvis and thoracic spine, treatment with intravenous zoledronic acid was considered appropriate following multidisciplinary discussion. Zoledronic acid remains the preferred first-line bisphosphonate across all three guidelines owing to its efficacy in achieving sustained biochemical remission.

**Key learning points:**

1. Persistent isolated elevation of alkaline phosphatase with normal liver function tests should prompt consideration of metabolic bone disease, particularly when bone-specific ALP is elevated.

2. Mild vitamin D deficiency should not be assumed to fully explain prolonged ALP elevation, especially when abnormalities persist despite correction of deficiency.

3. A detailed family history is important, as Paget’s disease has a recognised genetic predisposition and familial clustering.

4. Radionuclide bone scintigraphy is essential to define the extent of metabolically active disease following diagnosis and identify polyostotic involvement.

5. Intravenous zoledronic acid is the preferred first-line therapy when treatment is indicated, achieving sustained suppression of bone turnover and biochemical remission.

6. The management of asymptomatic Paget’s disease remains controversial. While UK guidance supports a symptom-directed approach, international recommendations suggest considering treatment in selected patients with active disease involving high-risk skeletal sites.

7. This case highlights the importance of avoiding diagnostic anchoring and maintaining a broad differential diagnosis when faced with persistent unexplained biochemical abnormalities.

